# Impact of home quarantine on physical fitness of school-aged children in Xi’an during COVID-19 lockdown: a cross-sectional study

**DOI:** 10.1186/s12889-024-18607-6

**Published:** 2024-04-25

**Authors:** Xinglu Li, Zijun Lu, Tao Liu, Yuliang Sun

**Affiliations:** School of Physical Education, Shaanxi Normal University, Xi’an, 710119 China

**Keywords:** Student’s physical fitness, Pandemic implications, Regional variations

## Abstract

**Background:**

The emergence of the COVID-19 pandemic has sparked unprecedented global challenges. This study intends to investigate changes in the physical fitness of students aged 6–22 during the COVID-19 pandemic and to assess how the pandemic lockdown period affected these markers.

**Methods:**

According to the National Student Physical Health Standard, a stratified cluster sampling method was used to evaluate the body shape, body function, and physical fitness of children and adolescents (*n* = 8092) in Xi’an from 2019 to 2021. This study uses SPSS 26.0 (IBM, Chicago, IL, USA) for data statistics and analysis. The connection between physical fitness and years was measured using the one-variable analysis in the general linear model (GLM). Independent t-tests were used to determine the sex (male/female) and area (urban/rural) differences.

**Results:**

During the lockdown period, Body Mass Index (BMI) and flexibility showed an upward trend, while aerobic, strength, speed, and endurance showed a downward trend. In addition to the BMI of middle and high school students, almost all indicators show significant sex differences. There are urban-rural differences in some indicators, such as chin-ups.

**Conclusion:**

During the pandemic of COVID-19, the physical fitness of children and adolescents in Xi’an did not change significantly, and there were slight differences among different grades. During the pandemic lockdown period, lifestyle changes and reduced outdoor activities for children and adolescents may be the reasons for the changing trend of various indicators.

## Background

The emergence of the COVID-19 pandemic has sparked unprecedented global challenges, profoundly impacting the lives and health of individuals worldwide [1]. The Chinese government has taken the most strict control measures to limit infection rates, which affected the adolescents and students more than others [2]. Several studies have found that the level of physical fitness of a country’s adolescents is related to the profile of the future adult population [3]. Prolonged school closures, online learning, and social restrictions have triggered huge changes in students’ lifestyles. These measures have inevitably influenced various aspects of daily life, particularly among children and adolescents. Understanding the effect of these measures on the physical fitness of students is crucial, especially within the unique socio-cultural context of China.

The restrictions on outdoor activities led to a decline in students’ physical activity levels during the COVID-19 [4]. The closure of schools and sports installations, the absence of extracurricular sports activities, and restrictions on socialization led to a significant reduction in the amount of time and opportunities for exercise for many students. The low level of physical activity may lead to a decline in students’ physical fitness [5], including decrease of muscle strength [6] and cardiorespiratory fitness [7].

The low level of physical fitness may symbolizes the deterioration of health [8]. The increase of sedentary time, which is caused by the online learning, may increase students’ health problems, such as postural problems, myopia, and obesity [9, 10]. Lack of face-to-face interaction with peers and teachers may also negatively impact students’ mental health [11–14]. In addition, changes in dietary habits may also have an impact on student fitness. During the pandemic, students may be more likely to favor convenience foods and snacks high in sugar and salt while neglecting balanced and nutritious eating habits, which may lead to malnutrition and weight problems [15].

Some studies investigated the trend of physical activities and nutritional status of teenagers during COVID-19 lockdown [16]. However, to our knowledge, there is little work that has investigated the trends in a large population-based cohort of students from 6 to 22 years old during the COVID-19 lockdown period in China. Compared to other studies, this study includes three rounds of tests, which were conducted before the outbreak, during home quarantine, and after the lifting of home quarantine. Aims to investigate the effects of the COVID-19 pandemic and associated lockdown measures on the physical fitness of children and adolescents aged 6–22 in Xi’an, China, spanning from 2019 to 2021. Additionally, we aim to explore potential disparities in physical fitness outcomes across sex and urban-rural divides. Based on previous studies, we hypothesised that the COVID-19 lockdown particularly negatively affected school-aged children and the body mass index of students should increase, while other physical fitness tests should show varying degrees of decline in the three years.

## Methods

### Participants

Data were obtained from the 2019, 2020, and 2021 waves of the Chinese National Survey on Students’ Constitution and Health (CNSSCH), a joint project of the Ministries of Education, Health, Science and Technology, the State Ethnic Affairs Commission, and the State Sports General Administration of the People’s Republic of China. The CNSSCH was conducted in 30 mainland provinces, autonomous regions, and municipalities among school-aged students aged 6–22 years to monitor trends in health, nutrition, and wellbeing [17]. According to the Chinese National Student Physical Health Standard (CNSPFS) (revised in 2014), a stratified cluster sampling method was used to evaluate the body shape, body function, and physical fitness of children and adolescents in Xi’an from 2019 to 2021 [2, 3, 18]. The school and the student that we tested each year were different. All schools are located in Xi’an, Shaanxi Province, China. And all schools were unified under the leadership of the Xi’an Government during the COVID-19.

**Fig. 1:**
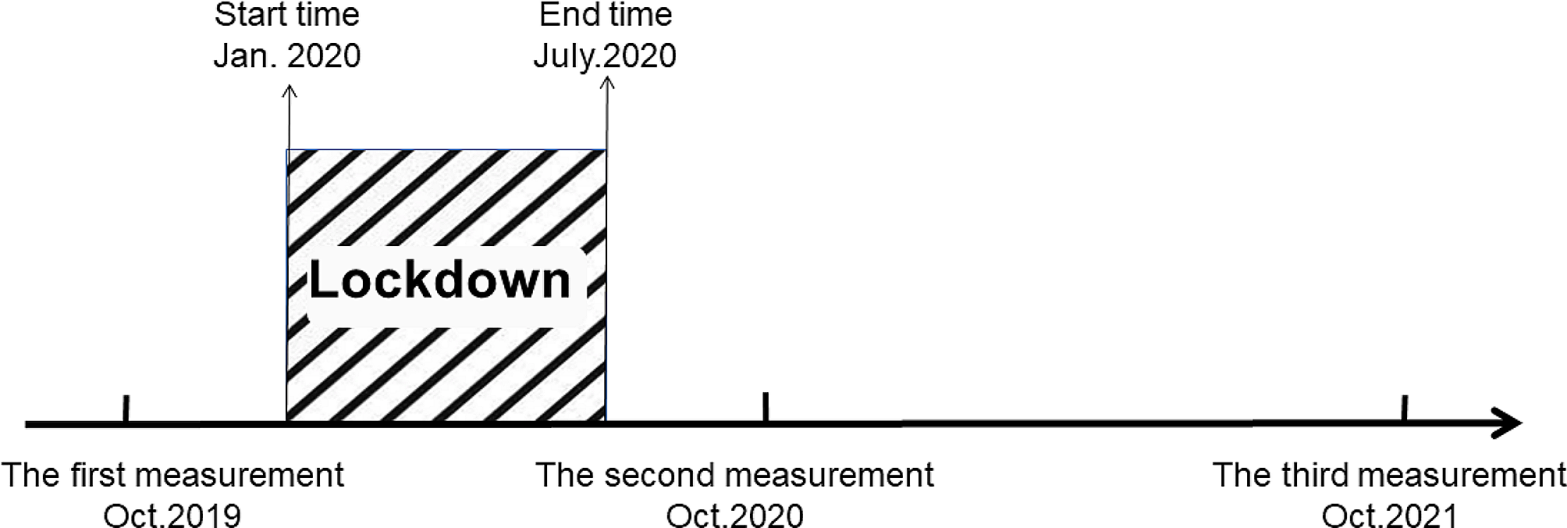
The time of physical fitness test and pandeminc lockdown in Xi’an

The participants were randomly selected in each grade. The number of students also changes with the real situation of the school. During the testing period, we only have a lower limit, not an upper limit, for the number of students tested. The sample size was calculated using G-power based on the following assumptions. Tail (s): One, Effect size d: 0.3; an error probability = 0.05; and power (1- β error probability) = 0.8. This gave a sample size of 278 [19]. Finally, 8092 students aged 6–22 years from primary school, middle school, high school and college were contained (Table 1), with physical fitness testing in the autumn of each year (Fig. 1). Primary school students are aged 6–11/12, middle school students are aged 12/13–14/15, high school students are aged 15/16–17/18, and college students are aged 18/19–21/22.

**Table 1:** Sample characteristics (*n* = 8092)

		Primary School (*n* = 2246)			Middle School (*n* = 1209)			High School (*n* = 1413)			College (*n* = 3224)		
		2019 *n* = 502	2020 *n* = 934	2021 *n* = 810	2019 *n* = 323	2020 *n* = 479	2021 *n* = 407	2019 *n* = 537	2020 *n* = 471	2021 *n* = 405	2019 *n* = 1497	2020 *n* = 901	2021 *n* = 826
Male	Urban	124	237	204	59	119	102	151	118	102	730	451	412
	Rural	115	230	204	93	120	101	97	116	101			
Female	Urban	134	237	204	70	120	102	191	119	102	767	450	414
	Rural	129	230	198	101	120	102	98	118	100			

During the testing period, the students were allowed to participate in physical education programms. Although they did not go out during the quarantine period, they still had the opportunity to engage in sports activities through online learning. Physical education major students, as well as athletes, students who are sick, and girls with special conditions will be excluded from the test with a doctor’s permission. During the testing process, students were healthy and tested negative for COVID-19. Two training sessions and a testing handbook were provided to all evaluators to minimize mistakes during the test. The local ethics committee approved the study at Shaanxi Normal University (201,916,001 2019-09). The informed consent was obtained from the students and their parents before each item was tested. Numerical coding of all students’ names was used to avoid disclosing their personal information.

### Assessment

The study used the CNSPFS (revised in 2014) as the test standard, which has received consistent approval from academics [20]. To reduce measurement error, the measurement instruments were calibrated before use. The test for each school was completed on a morning in October to reduce data deviation caused by different test times. Following the Chinese National Student Physical Fitness and Health Research handbook [21], the test projects include 50 m sprint, standing long jump, sit-and-reach, 800 m and 1000 m running, sit-ups, chin-ups, 50 m × 8 shuttle run, and 1 min rope skipping. According to previous studies, the tests were classified into five fitness batteries: endurance, strength, coordination, speed, and flexibility [22]. In addition, our study examined vital capacity and body mass index [16] (Table 2). In order to ensure the fluency of the test, the professional physical education teacher led the students in 30-minute warm-up activities before the test began. During the test, we recommend that height, weight, vital capacity, and visual acuity tests be tested first, followed by strength, flexibility, coordination, speed, and finally endurance.

**Table 2:** Test projects and test groups

Test		Primary School			Middle School	High School	College
		1–2 Grades	3–4 Grades	5–6 Grades			
Height(cm)/Weight(kg)		√	√	√	√	√	√
VC (ml)		√	√	√	√	√	√
Visual acuity		√	√	√	√	√	√
1 min Sit-ups	(male)	-	√	√	-	-	-
	(Female)	-	√	√	√	√	√
Chin-ups (male)		-	-	-	√	√	√
Standing long jump(cm)		-	-	-	√	√	√
50 m × 8 shuttle run(s)		-	-	√	-	-	-
800 m running (female)(s)		-	-	-	√	√	√
1000 m running (male)(s)		-	-	-	√	√	√
Sit-and-reach		√	√	√	√	√	√
50 m sprint(s)		√	√	√	√	√	√
1 min rope skipping		√	√	√	-	-	-

VC: Vital Capacity; √: Students are required to take part in this test; -: Students are not required to take part in this test

### Body Mass Index (BMI)

BMI is a standard index to measure the degree of body fat and whether it is healthy worldwide [23].

$$ \varvec{B}\varvec{M}\varvec{I}=\raisebox{1ex}{$\varvec{W}\varvec{e}\varvec{i}\varvec{g}\varvec{h}\varvec{t} \left(\varvec{K}\varvec{g}\right)$}\!\left/ \!\raisebox{-1ex}{${\varvec{H}\varvec{e}\varvec{i}\varvec{g}\varvec{h}\varvec{t}}^{2}\left(\varvec{m}\right)$}\right.$$

The BMI of children and adolescents aged 3–18 years was calculated and categorized according to the “Screening for overweight and obesity among school-age children and adolescents“ [24]. The BMI for adults is calculated as well as categorized according to the WHO (World Health Organization) standards. According to the WHO standard, body mass index includes overweight, obesity, medium, thinness, and severe thinness. Height and weight were used to evaluate students’ growth and development levels [25]. When measuring height, the subjects were barefoot and standing upright on the floor of the height meter (the upper limbs were naturally drooping, the heels were closed, and the toes were separated into 60 degrees). The heel, sacrum, and two scapular areas are in contact with the column, the trunk is naturally straight, the head is straightforward, and the upper edge of the tragus and the lower edge of the orbit are horizontal. Record results in centimeters to one decimal place int. When measuring body weight, subjects were barefooted, and male subjects wore shorts; female subjects wore shorts, short sleeves, or vests and stood in the center of the scale. The reading is in kilograms, accurate to one decimal place.

### Vital Capacity (VC)

VC refers to the total amount of gas exhaled after trying to inhale, which can reflect the potential ability of respiratory function in a certain sense [26]. Vital capacity was measured using the electronic spirometer. Electronic spirometer met specific technical criteria, ensuring an acceptable margin of error ≤ 3% [9]. During the test, the subjects take a deep breath, hold the breath, and exhale slowly to the mouth until they can no longer exhale to prevent inhaling from the mouth at this time and not inhale twice during the test [27]. Each subject was measured three times, and the maximum value was selected as the test result. In milliliters, no decimal number is retained.

### Strength

The strength test mainly includes upper limb strength, lower limb strength, and core strength. The chin-up test results reflected the upper limb strength of the subjects. During the test, the subject jumped up with both hands holding the bar, and both hands were overhanging at shoulder width. After resting, the two arms simultaneously pull the body (the body can not have additional action) and chin-ups to the lower jaw over the upper edge of the horizontal bar for completion [27]. Record the number of leads.

The sit-up test results reflect the core strength of the subjects. During the test, the subjects were supine on the pad, the legs were slightly separated, the knees were bent at an angle of about 90 degrees, and the two fingers were crossed behind the brain. Another partner pressed his ankle to secure his lower limbs. When the subjects sat up, two elbows touched or exceeded both knees to complete once. Two shoulder blades must touch the pad in a supine position. Record the number of completions within 1 min [28]. 

The standing long jump test results reflect the lower limb strength of the subjects. During the test, the subjects naturally stood on their feet separately. After standing on the take-off line, the toes should not step on the line. Two feet take off simultaneously; There must be no step or jump action. Measure the vertical distance from the trailing edge of the take-off wire to the nearest landing site [28]. Each person tried three times, recording the best one in centimeters.

### Endurance

The 50 m × 8 shuttle run and 800 m / 1000 m test reflect the development level of students’ endurance quality, especially the function of the cardiovascular and respiratory systems and muscle endurance. The subjects were tested in groups of at least two people standing up during the test. They start running when they hear the ‘run’ command. When the subject’s torso crosses the line, the timer stops. Record the test results in minutes and seconds, regardless of decimals [29]. 

### Flexibility

The range of motion that students can reach in the static state of the trunk, waist, hip, and other joints is measured by sitting forward flexion, which mainly reflects the extension and elasticity of joints, ligaments, and muscles in these parts and the development level of students’ physical flexibility. The subjects’ legs were straightened during the test, and the two feet were flat on the ground. The feet were separated by about 10–15 cm, and the upper body was bent forward. Before the arms were straightened, the cursor was gradually pushed forward with the fingertips in both hands until it could not be pushed forward. The inner edge of the longitudinal pedal plate of the tester is 0 point, negative inward and positive forward [28]. Record in centimeters as a unit, and retain a decimal number. Test twice, and take the best score.

The 1 min rope skipping test reflects students’ lower limb explosive force and body coordination ability. Test and record the number of rope skipping times in 1 min.

### Speed

The 50 m run tests the development level of students’ speed, sensitivity, and nervous system flexibility. The subjects were tested in groups of at least two people during the test. After hearing the command of ‘running,’ the subject starts to run, and the participant’s trunk reaches the vertical plane stop table of the finish line [29]. The researchers recorded the results in seconds, which were accurate to one decimal point.

### Visual acuity

Visual acuity (VA) is a measure of the ability of the eye to distinguish shapes and the details of objects at a given distance [30]. All the eye measurements were conducted and recorded by a professional eye care doctor. Position the student, sitting or standing, at a distance of 6 m from the chart. Test the eyes one at a time. Ask the student to cover one eye with a plain occluder, card or tissue. The student is asked to point in the direction the ‘legs’ of the E are facing. Record the VA for each eye in the student’s notes. Repeat the whole procedure for the second eye.

### Statistical analysis

SPSS 26.0 (IBM, Chicago, IL, USA) was used to analyze the data. The mean (M) and standard deviation (SD) of all variables were calculated. The data was verified through the Shapiro-Wilk test. Normality and homogeneity of variance for quantitative results were confirmed by the test (*p* > 0.05), and severe outliers were eliminated using a z-score cut-point of ± 3.0. The connection between physical fitness & nutritional status and years was measured using the one-variable analysis in the general linear model (GLM). Independent t-tests determined the sex (male/female) and area (urban/rural) differences. Each of the models had the covariate of age input.

## Results

### Trends in growth and development level

**Fig. 2:**
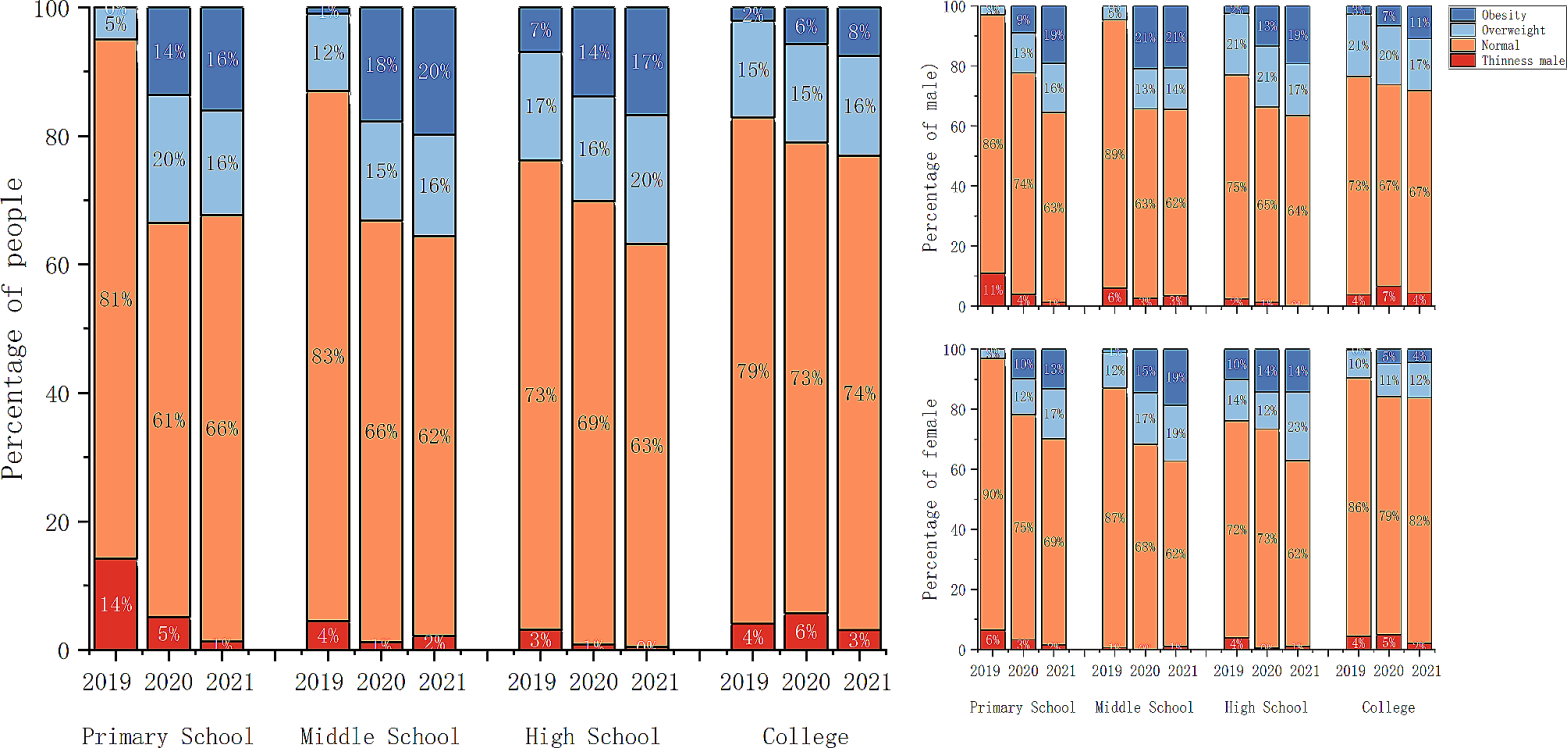
The growth and development level of participants from 2019–2021

There was a decline in thinness regarding development, whereas the group medium essentially stayed unchanged. However, there was a significant increase in overweight and obesity (Fig. 2). The number of overweight primary school students increased by 15%, during the COVID-19 lockdown period (2019–2020). The number of obesity primary school students increased by 16%, at middle school students increased by 19%, at high school students by 10% and at college students by 6%, during the lockdown period (2019–2021).

**Fig. 3:**
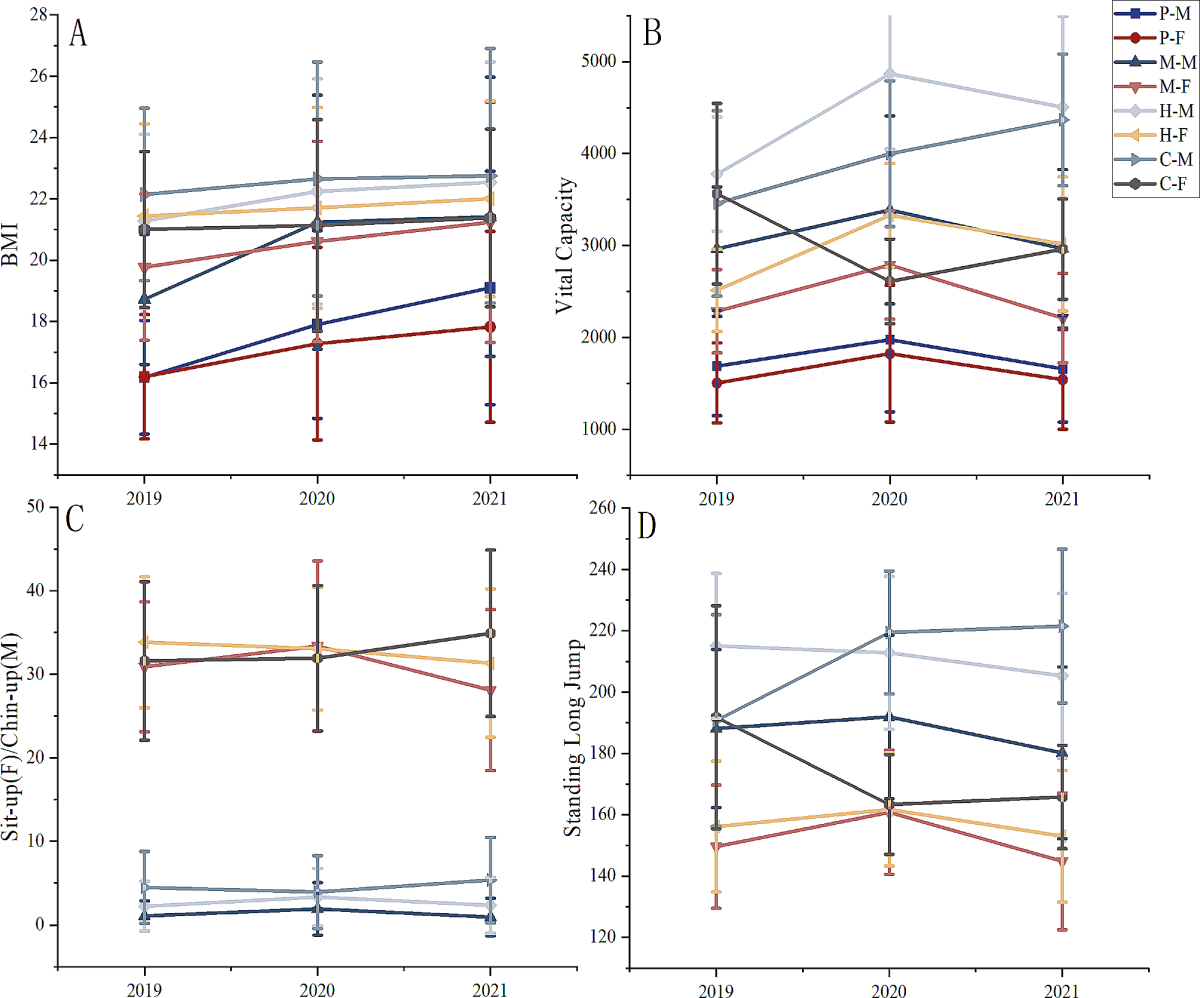
The trend of BMI, VC, standing long jump, sit-ups, and chin-ups.P is primary school, M is middle school, H is high school, C is college, M is male, and F is female

Between 2019 and 2021, students’ BMI in middle and high school rose. Nevertheless, female high school students and college students saw this tendency flatten. The rise between primary and middle school was more pronounced compared to previous growth periods. Compared to the growth from 2020 to 2021, the increase from 2019 to 2020 was greater (Fig. 3A).

In primary, middle, and high schools, the trend of essential capacity, which climbed from 2019 to 2020 while declining from 2020 to 2021, deserves to be recognized. On the other hand, one of the female college students’ numbers climbed in 2020 and declined in 2019, respectively. From 2019 to 2021, the trend of male college students grew (Fig. 3B).

### Trends in physical fitness

There was no significant difference in the grades of chin-ups and sit-ups (Fig. 3C). The middle and high school students’ standing long jump scores decreased and then increased over the three years. The opposite was true for female college students. The standing long jump scores of male college students continued to improve over the three years (Fig. 3D).

**Fig. 4:**
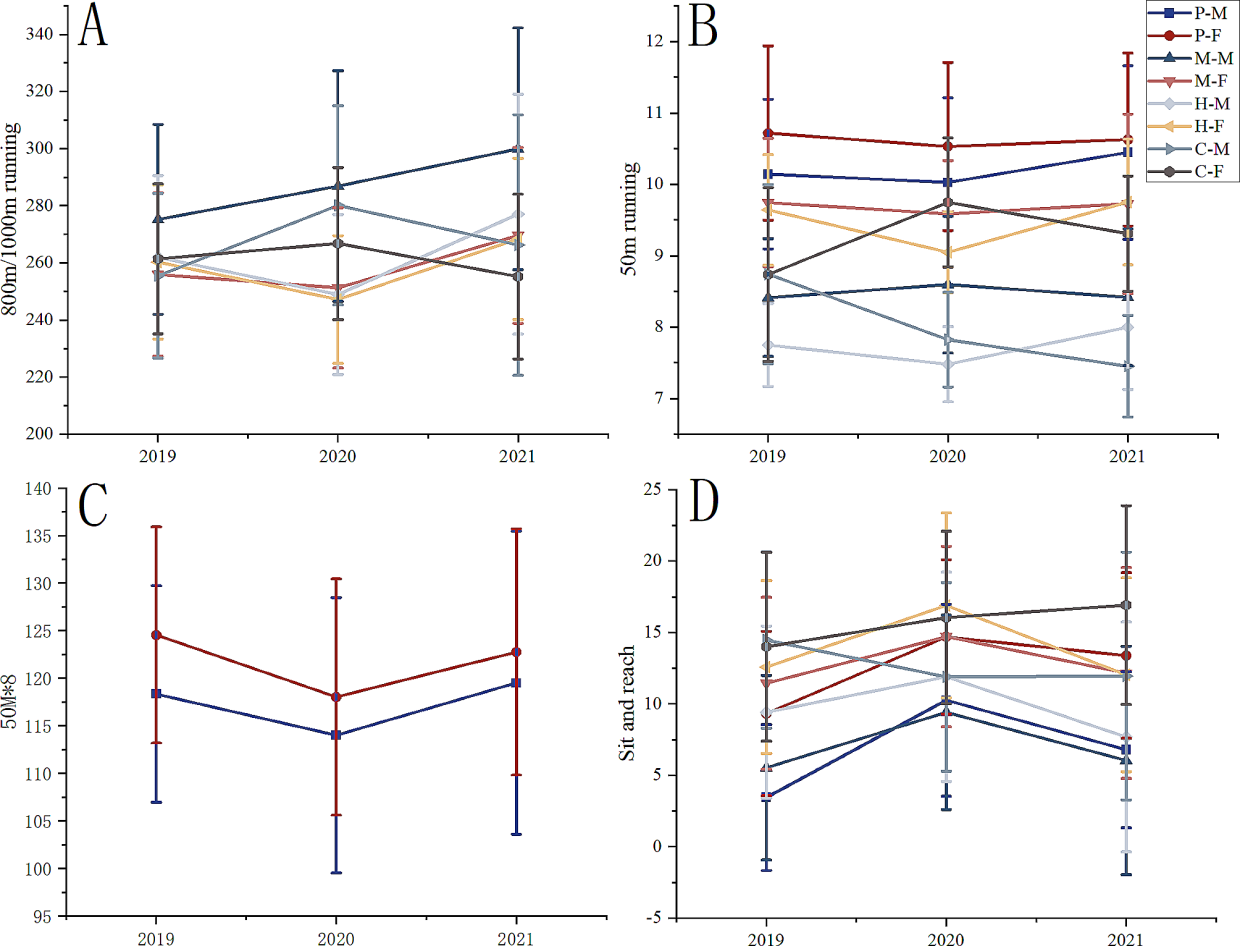
The trend of 800 m/1000m running, 50 m sprint, Sit-and-reach 50 m × 8 shuttle run

The 800 m running scores of all female students followed the same trend as the standing long jump, decreasing and then increasing. In contrast, the 1000 m running scores of all male students continued to decrease over three years (Fig. 4A). The scores of the 50 m sprint have a downward trend from 2019 to 2021 in all participants except college females. The opposite was noted for college females (Fig. 4B). The scores of 50 m × 8 shuttle run for primary school students decreased and then increased (Fig. 4C).

The sit-and-reach scores increased from 2019 to 2020 and decreased from 2020 to 2021 for all participants except college males. The opposite was noted for college males (Fig. 4D).

**Fig. 5:**
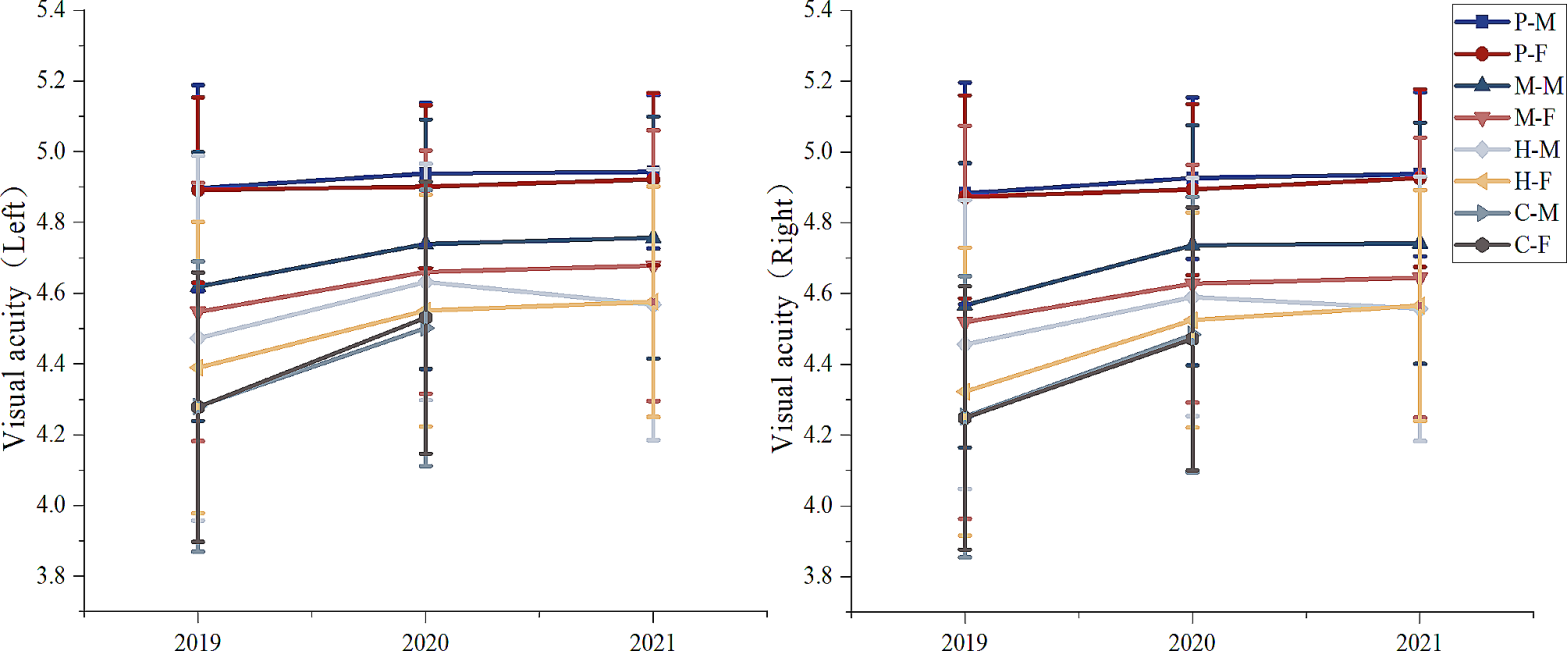
The trend of visual acuity (left and right)

The results of visual acuity scores (left and right) indicate that there was a slight decrease in visual acuity among all students over the three years, but the change was not significant. (Fig. 5).

### Difference in sex and region

Except for the BMI of middle and high school children, the indicators demonstrated a significant sex difference (*p* < 0.05), as shown in Table 3. Males were significantly better than females in vital capacity, lower limb strength and speed. Females were significantly better than males in flexibility.

**Table 3:** Physical fitness differences in sex

	Primary School *n* = 2246						Middle School *n* = 1209						High School *n* = 1413						College *n* = 3224					
	Male M(SD)	Female M(SD)	T	*p*	D	ES	Male M(SD)	Female M(SD)	T	*p*	D	ES	Male M(SD)	Female M(SD)	T	*p*	D	ES	Male M(SD)	Female M(SD)	T	*p*	D	ES
BMI (kg/m^2^)	17.9 (3.33)	17.2 (2.97)	5.601	< 0.001	0.24	S	20.7 (4.05)	20.6 (3.39)	0.329	0.743	0.02	N	22.0 (3.51)	21.7 (3.15)	1.674	0.094	0.09	N	22.4 (3.50)	21.1 (2.91)	11.559	< 0.001	0.41	M
VC (mL)	1796.8 (682.36)	1648.2 (629.57)	5.365	< 0.001	0.23	S	3134.1 (910.81)	2455.8 (584.74)	15.349	< 0.001	0.89	L	4364.7 (937.32)	2917.8 (673.35)	33.148	< 0.001	1.78	L	3845.6 (959.97)	3146.3 (870.01)	21.657	< 0.001	0.76	M
Standing Long Jump (cm)			-	-			186.9 (27.34)	152.4 (22.02)	24.162	< 0.001	1.39	L	211.4 (25.36)	157.1 (20.77)	43.878	< 0.001	2.35	L	206.8 (32.27)	177.3 (30.88)	26.495	< 0.001	0.94	L
Sit-ups ^a^	29.4 (9.11)	27.9 (8.87)	3.415	< 0.001	0.17	N		30.9 (9.63)	×	×				32.9 (8.04)	×	×				32.8 (9.48)	×	×		
Chin-ups			-	-	-	-	1.4 (2.61)		×	×			2.7 (3.29)		×	×			4.6 (4.65)		×	×		
Sit-and-reach (cm)	7.5 (6.50)	12.9 (6.00)	20.661	< 0.01	0.87	L	7.3 (6.36)	13.0 (6.75)	-14.01	< 0.001	0.81	L	9.8 (7.31)	13.8 (6.74)	-10.918	< 0.001	0.58	M	13.1 (7.13)	15.3 (6.66)	-9.132	< 0.001	0.32	M
50m × 8 shuttle run (s) ^b^	10.2 (1.18)	10.6 (1.20)	-5.866	< 0.001	0.36	S			-	-					-	-					-	-		
1 min rope skipping	102.1 (36.07)	108.4 (32.71)	-4.319	< 0.001	0.18	N			-	-					-	-					-	-		
50m sprint (s)	10.2 (1.18)	10.6 (1.20)	-7.993	< 0.001	0.34	S	8.5 (0.93)	9.7 (0.98)	21.585	< 0.001	1.24	L	7.7 (0.700	9.5 (0.80)	43.722	< 0.001	2.32	L	8.1 (1.14)	9.2 (1.13)	25.406	< 0.001	0.90	L
800m running (female)(s)			-	-				258.7 (30.19)	×	×				258.3 (27.22)	×	×				261.3 (27.67)	×	×		
1000m running(male)(s)			-	-			288.5 (40.52)		×	×			262.0 (34.77)		×	×			268.3 (38.68)		×	×		

BMI: body mass index; VC: Vital Capacity; -: Students are not required to take part in this test; ×: The test was attended by male or female students only, and no sex differences were compared; a: Primary school students in grades 3 to 6 were tested sit-ups; b: Primary school students in grades 5 and 6 were tested 50 m × 8 shuttle run; M(SD): Mean (Standard Deviation); ES, effect size; N, no effect: d < 0.2; S, small effect: 0.2 ≤ d ≤ 0.5; M, medium effect:0.5 ≤ d ≤ 0.8; L, large effect: d > 0.8

Most indicators might also be used to identify the regional variation (Table 4), including the vital capacity, the grades of 50 m×8 shuttle run, the quantity of 1 min rope skipping of students in primary school, the BMI, VC, Standing Long Jump, Chin-ups (male), Sit-and-reach, and 50 m sprint of students in middle school, the VC, the standing long jump, the chin-ups(male), the sit-ups(female) and the grades of 800 m/1000m running of students in high school.

**Table 4:** Physical fitness differences in the region

	Primary School *n* = 2246						Middle School *n* = 1209						High School *n* = 1413					
	Urban M(SD)	Rural M(SD)	T	*p*	D	ES	Urban M(SD)	Rural M(SD)	T	*p*	D	ES	Urban M(SD)	Rural M(SD)	T	*p*	D	ES
BMI (kg/m^2^)	17.6 (3.28)	17.6 (3.07)	0.302	0.762	0.01	N	21.0 (3.77)	20.2 (3.60)	3.679	< 0.001	0.21	S	21.7 (3.30)	22.0 (3.37)	-1.378	0.168	0.08	N
VC (mL)	1922.9 (667.88)	1514.8 (584.39)	15.425	< 0.001	0.65	M	2894.8 (769.28)	2694.0 (878.48)	4.236	< 0.001	0.24	S	3592.3 (1061.42)	3652.6 (1118.69)	-1.036	0.3	0.06	N
Standing Long Jump (cm)			-	-	-		163.1 (28.63)	175.0 (30.49)	-6.953	< 0.001	0.40	S	186.3 (35.15)	179.7 (35.97)	3.48	< 0.01	0.19	N
Sit-ups ^a^	28.3 (8.87)	29.0 (9.16)	-1.383	0.167	0.07	N	31.2 (9.26)	30.7 (9.96)	0.634	0.526	0.05	N	34.3 (7.90)	31.0 (7.85)	5.586	< 0.001	0.42	S
Chin-ups(male)			-	-			1.0 (1.92)	1.7 (3.06)	-3.612	< 0.001	0.29	S	2.1 (3.28)	3.3 (3.17)	-4.853	< 0.001	0.38	S
Sit-and-reach (cm)	10.5 (6.62)	10.0 (7.02)	1.862	0.063	0.08	N	10.2 (7.48)	10.2 (7.73)	-26.983	< 0.01	0.00	N	12.2 (7.75)	11.5 (6.72)	1.731	0.084	0.09	N
50 m × 8 shuttle run (s) ^b^	121.8 (14.16)	124.3 (15.62)	-2.781	< 0.05	0.17	N			-	-					-	-		
1 min rope skipping	110.6 (34.9)	99.7 (33.37)	7.564	< 0.001	0.32	S			-	-					-	-		
50 m sprint (s)	10.4 (1.23)	10.4 (1.19)	0.302	0.762	0.04	N	9.0 (1.03)	9.2 (1.20)	3.679	< 0.001	0.14	N	8.6 (1.11)	8.7 (1.21)	-1.378	0.168	0.08	N
800 m running (female)(s)			-	-			257.9 (28.26)	259.4 (31.89)	-45.074	< 0.01	0.05	N	260.2 (25.68)	255.8 (28.92)	-1.553	0.121	0.16	N
1000 m running(male)(s)			-	-			290.4 (40.35)	286.8 (40.67)	-0.622	0.534	0.09	N	262.0 (31.52)	262.0 (38.30)	2.149	0.032	0.00	N

BMI: body mass index; VC: Vital Capacity; -: Students are not required to take part in this test; ×: The college is not divided into regions and does not compare urban and rural differences; a: Primary school students in grades 3 to 6 were tested sit-ups; b: Primary school students in grades 5 and 6 were tested 50 m × 8 shuttle run. M(SD): Mean (Standard Deviation); ES, effect size; N, no effect: d < 0.2; S, small effect: 0.2 ≤ d ≤ 0.5; M, medium effect:0.5 ≤ d ≤ 0.8; L, large effect: d > 0.8

## Discussion

This study shows that BMI and flexibility increased during lockdowns, while aerobic, strength, speed, and endurance are on a downward trend. With the exception of BMI for middle school and high school students, most indicators showed significant sex and region differences; The results basically approved our hypnotizes, except for the flexibility indicators.

This study shows that the overweight and obese students increased during the COVID-19 lockdown. This is consistent with the results of previous studies. This may be related to a reduction in dietary activity and physical activity [31–32]. However, some studies may have already indicated historical data of pre-pandemic China on students’ obesity rates and BMI, showing a gradually increasing trend [33–37]. Along with the success of poverty eradication, a convergence of urban and rural lifestyles has been occurring in China [38], which may improve the nutritional status of students. Therefore, the factors of the increased BMI may be complicated, which deserves to be focused in the future.

The erobic fitness (800 m run/1000m run) and speed (50 m run) of the students decreased in this study, consistent with previous studies [39]. The study showed that young adults experienced a 3.84% drop in 1000 m performance and a 12.69% drop in chin-ups after two months of lockdown [40]. There was no significant difference in chin-ups and sit-ups performance. The result is inconsistent with previous studies that showed improvements in both female sit-ups and male chin-ups [41]. The upper strength may be influenced better by growth, which means the effect of lockdown may be covered.

Differ from other indicators, the flexibility and lower limb strength improved during the COVID-19 lockdown, which is slightly disapproved initial hypothesis. This indicated that the lockdown may delayed the growth of different physical fitness indicators to varying degrees. Gonzalez JW et al. also found an increasing trend in lower limb strength during the COVID-19 lockdown [33]. The limited exercise space at home may contribute to fewer exercise patterns to choose. During the pandemic, yoga and skipping rope became more common sports due to the restrictions of activity venues [42–43]. Exercises that increase core strength have been demonstrated to enhance flexibility [40]. Practicing yoga affects students’ flexibility qualities clearly [44].

We found significant differences between almost male and female students in almost all physical fitness. Males were significantly better than females in vital capacity, lower body strength and speed. Males generally have higher levels of muscle mass [45, 46] and vital capacity [47] than females, which can give them an advantage in sports that require strength and endurance. This is in line with other studies reporting normative values [48–50]. For example, a study described that boys significantly outperformed girls in cardiorespiratory endurance, speed, and lower limb strength [49]. Additionally, females tend to have higher body fat [51], which make it more challenging to perform sports that demand speed and power. Females were significantly better than males in flexibility. This finding was supported by past reports that used sit and reach test [52–54]. A study investigating 7–14 year old children reported better flexibility in females than males [50]. Another study indicated that superior flexibility was reported in 6–12 years old girls compared with their same age male counterparts [48].

We found significant differences between almost urban and rural students in almost physical fitness. Urban students in middle schools have significantly higher BMI than rural students. A study found that the overweight was more prevalent in urban middle schools [55], while another study found that children in rural areas were 26% more likely to be obese compared to children in urban areas [56]. In terms of dietary structure, urban students are more likely to be exposed to high-energy, high-sugar foods, which can contribute to health problems such as obesity and diabetes [57].

Urban students were better than their rural counterparts in most of the physical fitness indicators. According to findings from studies conducted in Ecuador [58] and Mexico [59], it was observed that children and adolescents residing in urban areas exhibited superior levels of cardiorespiratory fitness and muscular strength compared to those in rural areas. Conversely, conflicting results emerged from research conducted in Austria [60], Spain [61], and Turkey [62], where urban dwelling was linked to increased body weight and decreased physical fitness levels. Rural areas have fewer accessible sports facilities. This can result in reduced opportunities for regular exercise [63]. Differences in living habits between urban and rural students can also contribute to various health outcomes, such as diet, work and rest patterns, and leisure activities can all influence overall health and well-being [64].

### Strengths and limitation

Strengths of this study include: Participants consisted of school-age children and adolescents aged 6–22 years, and the measurement period included two blockades of COVID-19, which could explain the trends in student fitness under lockdown over three years.

### Limitations of this study include

Other potential variables, such as physical activity activity levels, eating habits, home address, and parental literacy, were not examined, which could explain the results better. In addition, there was ambiguous information about whether participants were infected with Covid-19, which could have affected the test results and required special attention.

Although we considered sex and regional variables, we did not delve into their interaction. A more comprehensive study of the interaction between these factors could provide deeper insights into explaining the patterns and trends of physical fitness changes. Therefore, future research should focus on these aspects to make our study more comprehensive and reliable.

While this study did not including student athletes, we acknowledge the importance of focusing on this subgroup in future research. This emphasis will undoubtedly contribute to a more comprehensive understanding of their characteristics and behaviors.

## Conclusions

This study found that a period of lockdown had different degrees of impact on physical fitness testing for school-age children, which may be affected by lifestyle changes. Students at home intake more energy but consume less energy, resulting in varying degrees of obesity. Decreased physical activity time, increased sedentary time, increased screen time, and other reasons caused changes in students’ physical fitness indicators. Therefore, in the future, schools should improve the fun of physical education classes and cultivate college students’ interest in physical exercise; Students’ motivation to participate in physical exercise should be improved so that students can actively participate in physical exercise; Ensure that students develop positive social attitudes and behaviors that lead to a healthy lifestyle.

## Data Availability

No datasets were generated or analysed during the current study.

## Abbreviations

GLM: General linear model
BMI: Body Mass Index
CNSSCH: Chinese National Survey on Students’ Constitution and Health
CNSPFS: Chinese National Student Physical Health Standard
WHO: World Health Organization
VA: Visual acuity
VC: Vital Capacity
COVID-19: Corona Virus Disease 2019
M: Mean
SD: Standard Deviation
